# The Role of Vti1a in Biological Functions and Its Possible Role in Nervous System Disorders

**DOI:** 10.3389/fnmol.2022.918664

**Published:** 2022-05-27

**Authors:** Fajuan Tang, Jiali Fan, Xiaoyan Zhang, Zhuan Zou, Dongqiong Xiao, Xihong Li

**Affiliations:** ^1^Department of Emergency, West China Second University Hospital, Sichuan University, Chengdu, China; ^2^Key Laboratory of Birth Defects and Related Diseases of Women and Children, Ministry of Education, Sichuan University, Chengdu, China

**Keywords:** Vti1a, N-ethylmaleimide-sensitive factor attachment protein receptor protein, neurosecretion, spontaneous synaptic transmission, gene fusion

## Abstract

Vesicle transport through interaction with t-SNAREs 1A (Vti1a), a member of the N-ethylmaleimide-sensitive factor attachment protein receptor protein family, is involved in cell signaling as a vesicular protein and mediates vesicle trafficking. Vti1a appears to have specific roles in neurons, primarily by regulating upstream neurosecretory events that mediate exocytotic proteins and the availability of secretory organelles, as well as regulating spontaneous synaptic transmission and postsynaptic efficacy to control neurosecretion. Vti1a also has essential roles in neural development, autophagy, and unconventional extracellular transport of neurons. Studies have shown that Vti1a dysfunction plays critical roles in pathological mechanisms of Hepatic encephalopathy by influencing spontaneous neurotransmission. It also may have an unknown role in amyotrophic lateral sclerosis. A *VTI1A* variant is associated with the risk of glioma, and the fusion product of the *VTI1A* gene and the adjacent *TCF7L2* gene is involved in glioma development. This review summarizes Vti1a functions in neurons and highlights the role of Vti1a in the several nervous system disorders.

## Introduction

Vesicle trafficking is an essential process in neuronal information transmission and is regulated by various regulators, such as the N-ethylmaleimide-sensitive factor attachment protein receptor (SNARE) protein family and Munc18-1 (Tang et al., 2021; Koike and Jahn, 2022). Membrane fusion mediated by the SNARE protein family is critical for vesicle trafficking (Mima, 2019). Based on their different functions in membrane fusion, SNARE proteins are divided into transport vesicle-associated SNAREs [v-SNAREs, such as Synaptobrevin (Syb)/vesicle-associated membrane protein (VAMP)] and SNAREs associated with target membrane localization [t-SNAREs, such as SNAP25 and Syntaxin (Stx)]; they spontaneously form complexes to carry out their functions (Katz and Brennwald, 2000). Furthermore, SNAREs are differentiated into Q-SNAREs and R-SNAREs based on the polar side chains associated with each SNARE central layer that contain highly conserved glutamine (Q) or arginine (R) residues (Kloepper et al., 2007). There are three Q-SNARE subfamilies, Qa, Qb, and Qc, and each SNARE complex contains one R-SNARE and three Q-SNAREs (Sutton et al., 1998). Currently, more than 30 SNARE proteins can be combined into unique complexes that drive specific membrane fusions (Bock et al., 2001).

The SNARE complex catalyzes the fusion of synaptic vesicles with the presynaptic membrane (Chen et al., 2021; Sauvola and Littleton, 2021). The canonical neuronal SNARE complex that mediates this process consists of the vesicular protein VAMP2 and the plasma membrane-associated proteins Syntaxin-1 and SNAP25 (Sudhof, 2004). These proteins are necessary for normal synaptic transmission, but some types of transmission are less dependent on these proteins. For example, deletion of the VAMP2 or SNAP25 genes in mice results in severely impaired stimulation-evoked neurotransmitter release (Liu et al., 2019). However, among the forms of spontaneous neurotransmitter release, a deficiency of VAMP2 or SNAP25 is less consequential (Ramirez and Kavalali, 2012; Liu et al., 2019). These results indicate the existence of other non-canonical SNARE proteins that are involved in synaptic vesicle fusion and could preferentially support spontaneous synaptic transmission (Kononenko and Haucke, 2012). VAMP7 and vesicle transport *via* interaction with t-SNARE homolog 1A (Vti1a) are synaptic vesicle proteins that cause spontaneous neurotransmitter release and regulate neuronal activity by mediating spontaneous neurotransmission (Ramirez and Kavalali, 2012; Crawford et al., 2017).

## Overview of VTI1A

The Vti protein was originally discovered in yeast (Fischer von Mollard and Stevens, 1998). In mammals, the Vti protein has two orthologs, Vti1a and Vti1b, which are widely expressed in tissues (Kreykenbohm et al., 2002). Vti1a is primarily localized to the *trans-*Golgi network (TGN) in cells, while Vti1b is localized to late endosomes (Kreykenbohm et al., 2002). In neurons, Vti1a is also localized to the cell body and presynaptic terminals, and splice variants of this protein are enriched in purified synaptic vesicles (Antonin et al., 2000b; Takamori et al., 2006). Vti1a and Vti1b appear to have different functions. For example, Vti1a plays an essential role in insulin-stimulated glucose transport (Bose et al., 2005); and regulates exocytosis in adrenal chromaffin cells (Walter et al., 2014). The loss of Vti1a impairs exocytosis, while cells lacking Vti1b do not exhibit any secretory defects (Walter et al., 2014). Vti1a and vti1b may have key overlaps in certain functions. For instance, deletion of either vti1a or vti1b alone is tolerable in mice, whereas deletion of both results in extensive neurodegeneration and perinatal lethality (Kunwar et al., 2011). Additionally, in Vti1a/b double-deficient neurons, impaired synaptic density, decreased secretion efficiency, and Golgi cargo accumulation have been observed, which could be rescued by the expression of Vti1a or Vti1b (Emperador-Melero et al., 2018).

## Gene and Protein Structure of VTI1A

*VTI* genes are highly conserved, and in the published genomics data, only six loss-of-function mutations have been reported, of which three are *VTI1A* (Lek et al., 2016). The *VTI1A* gene is located on chromosome 10q25.2 (Zhang et al., 2018). Although 14 heterozygous deletions or duplications in *VTI1A* and two duplications in *VTI1B* have been reported in patients diagnosed with neurodevelopmental delay and intellectual disability, these numbers are lower compared to other SNARE genes (Firth et al., 2009). However, studies have reported that the fusion product between the human *VTI1A* gene and adjacent genes plays an important role in the occurrence of cancer (Zhang et al., 2018). Furthermore, *VTI1A* variants are associated with cancer risk (Su et al., 2015). For example, in lung cancer caused by smoking, the hypomethylation of *VTI1A* variants is involved in carcinogenesis (Gao et al., 2016). Interestingly, hypomethylation of *VTI1A* variants also is involved in the development of hypertriglyceridemia (Guardiola et al., 2022).

**FIGURE 1:**
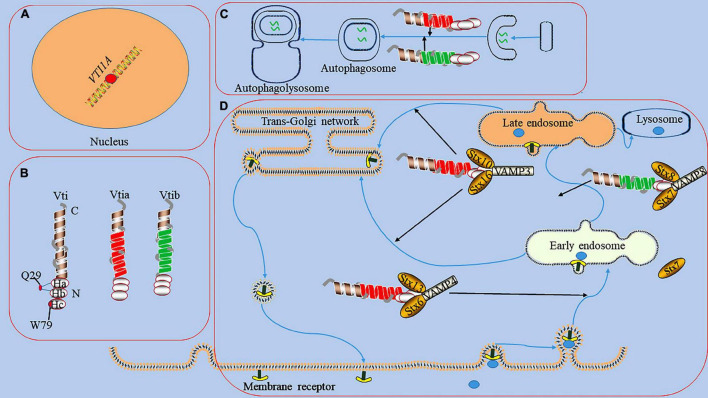
The functions of Vti1a in endosomal circulation and the formation of autophagosome. panel **(A)**, it represents the dot of *VTI1A* on Mutations/variations; the mutations/variations and gene fusions of VTI1A (red dots) in glial cells may be oncogenic. Panel **(B)**, it shows the domains and structures of Vti1a and Vti1b, a Vti protein contains a C-terminal transmembrane domain, a Qb-SNARE motif, and an N-terminal Habc domain; the Habc domain exists 2 mutants (Q29R localized in the loop between Ha and Hb and W79R localized on Hc); there has a low sequence homology between Vti1a and Vti1b. Panel **(C)**, it displays the process of the formation of autophagosome; Vti1a and Vti1b mediates autophagosome maturation. Panel **(D)**, it reveals endosomal circulation; Vti1a forms a SNARE complex with Syntaxin (Stx)10, Stx16, and VAMP3 to mediate the transport of related substances from the endosome to the Golgi apparatus. Vti1a co-immunoprecipitates with VAMP4, Stx6, and Stx16 in early circulating endosomal transport. Vti1b forms a complex with Stx7, Stx8, and VAMP8 and plays a role in late endosomal fusion.

It exists a low sequence homology between Vti1a and Vti1b (31–33% homology) (Emperador-Melero et al., 2019). All Vti proteins contain a C-terminal type II transmembrane domain, a Qb-SNARE motif, and an N-terminal triple helix Habc domain (Figure 1; Antonin et al., 2002). The Habc domain of Vti proteins has multiple functions, including the recruitment of tethered proteins and regulators and the correct classification of SNARE proteins (Gossing et al., 2013). Its abnormality can affect the stability of protein structure and impair the function. In carboxypeptidase Y (CPY) trafficking in yeast, the folded Habc domain is critical for proper CPY trafficking and late endosomal SNARE complex assembly (Conibear and Stevens, 1998). Two temperature-sensitive mutants (Q29R and W79R) in the Habc domain of Vti have been found to cause CPY sorting defects (Gossing et al., 2013). Vti1a is a vesicle-transporting V-SNARE protein considered a Qb-SNARE due to the central layer of aspartate residues which acts similar to glutamine (Zwilling et al., 2007). It regulates cellular secretion and has important physiological functions in the nervous system (Tang, 2020).

## Physiological Functions of VTI1A in the Nervous System

Glial cells and neurons are the main secretory cells in the nervous system, and SNAREs are the important regulators (Verkhratsky et al., 2016; Karim and Brett, 2018; Vilcaes et al., 2021). Lysosome exocytosis in astrocytes is mainly dependent on VAMP7, and the downregulation of VAMP7 expression inhibits the fusion of ATP storage vesicles and the propagation of ATP-mediated intercellular Ca^2+^ wave (Verderio et al., 2012). A study had found that Vti1a is closely related to glioma (Wang et al., 2017). Although there is an evidence that Vti1a plays a pivotal role in intracellular signaling by regulating the secretion of intracellular organelles such as Golgi apparatus, lysosomes, and endosomes (Emperador-Melero et al., 2019). But there is no direct evidence for how Vti1a works in glial cells. Vti1a appears to carry out actions in neurons, including regulation of neurosecretion by affecting upstream neurosecretory events, spontaneous synaptic transmission, and postsynaptic efficacy, as well as regulation of neural development, autophagic activity, and unconventional extracellular transport of neurons (Ganley et al., 2008; Flowerdew and Burgoyne, 2009; Kunwar et al., 2011; Lu et al., 2013; Crawford et al., 2017; Emperador-Melero et al., 2018).

### Regulation of Neurosecretion

**FIGURE 2:**
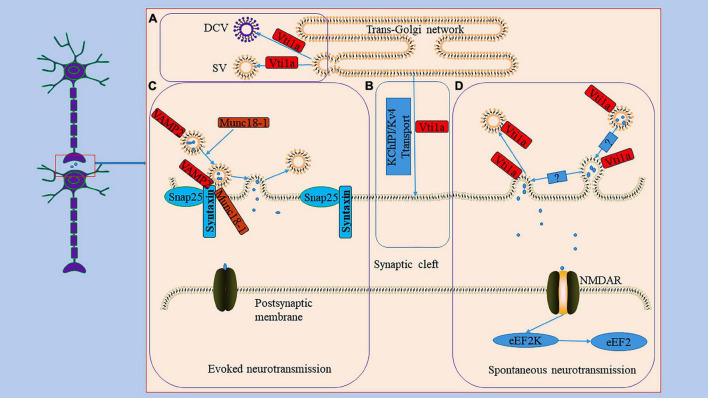
The role Vti1a in spontaneous transmission. Panel **(A)**, it reveals the generation of DCV and SV in TGN; Vti1a is mainly localized to TGN to regulate the secretion of SV and DCV. Panel **(B)**, it shows an unconventional exocrine pathway; Vti1a transports KChIP1/Kv4 through an unconventional exocrine pathway. Evoked neurotransmission and spontaneous neurotransmission are the two main ways of synaptic transmission. Panel **(C)**, it represents the process of the evoked neurotransmission; the synaptic vesicle protein VAMP2 forms a SNARE complex with SNP25 and Syntaxin and mediates vesicle trafficking in evoked neurotransmission under the regulation of Munc18-1. Panel **(D)**, it displays the process of the spontaneous transmission; Vti1a drives synaptic vesicle activity to mediate spontaneous transmission with an unclear mechanism to activate NMDAR to activate eEF2K to dephosphorylate eEF2.

The Vti1a protein regulates neuronal secretion by controlling exocytosis proteins and availability of secretory organelles (Ganley et al., 2008; Emperador-Melero et al., 2018). Neuronal communication primarily depends on the secretion of signaling molecules carried by synaptic vesicles (SVs) and dense core vesicles (DCVs) (Emperador Melero et al., 2017). SVs store neurotransmitters and are locally recovered after exocytosis (Haucke et al., 2011; Jahn and Fasshauer, 2012). DCVs are continuously produced in the TGN and mainly stores neuropeptides and neurotrophic factors (Wong et al., 2012; Moro et al., 2021). Studies have indicated that a single deletion of Vti1a or combined deletion of Vti1a and Vti1b decreases SV and DCV release related to the Ca^2+^-dependent exocytosis of SV and DCV regulated by Vti1a (Figure 2; Walter et al., 2014; Crawford et al., 2017). In Vti1a/b double knockout neurons, SV and DCV secretion was reduced, and the exocytotic proteins SNAP25 and Munc13-1 were decreased to a similar extent; overexpression of Vti1a or Vti1b rescued this change (Emperador-Melero et al., 2018).

Vti1a also cooperates with several SNAREs associated with the TGN and endosomes to mediate vesicular trafficking processes (Figure 1; Simonsen et al., 1998; Antonin et al., 2000a; Wendler and Tooze, 2001; Ganley et al., 2008). For example, Vti1a co-immunoprecipitates with VAMP4, Stx6, and Stx16 and assembles into a structurally conserved SNARE complex that mediates early, circulating endosomal transport of Shiga toxin and TGN46 (Simonsen et al., 1998; Wendler and Tooze, 2001). In addition, Vti1a forms a SNARE complex with Stx10, Stx16, and VAMP3 to mediate mannose 6-phosphate receptor (MPR) transport from endosomes to the Golgi (Ganley et al., 2008). Vti1b also plays a role in late endosomal fusion involving Stx7, Stx8, and VAMP8 (Antonin et al., 2000a). Vti1a/b-deficient neurons exhibit impaired synaptic density, secretion efficiency, Golgi size, and Golgi cargo accumulation (Emperador-Melero et al., 2018). Vti1a also may indirectly affect neurotransmitter transmission by influencing vesicle formation at the TGN as well as DCV generation (Walter et al., 2014).

It is known that action potential-evoked neurotransmitter release is a central process in the synaptic transmission of information (Rama et al., 2018). Furthermore, studies have demonstrated that spontaneous vesicle fusion plays a vital role in this process and is autonomous and heterogeneous in interneuron communication (Figure 2; Kavalali, 2015; Astacio et al., 2022). Spontaneous neurotransmitter release is not related to neuronal electrical activity but is equally involved in regulating synaptic stability and plasticity (Frank et al., 2006). Currently, it is debated whether the spontaneous neurotransmitter release has a dedicated pool of vesicles. For example, the same vesicles were involved in active and spontaneous release in cultured neurons from mouse (Wilhelm et al., 2010). However, in another study, both evoked and spontaneous vesicle pools were generated in rat hippocampal neurons (Fredj and Burrone, 2009). Vti1a is thought to regulate spontaneous fusion vesicles, driving synaptic vesicle activity to mediate spontaneous neurotransmitter transmission (Hoopmann et al., 2010; Kononenko and Haucke, 2012; Ramirez and Kavalali, 2012). An evidence suggests that endosome sorting is required for the production of exocytosing synaptic vesicles (Hoopmann et al., 2010). And Vti1a regulates the endosome sorting process (Emperador-Melero et al., 2019). In neurons from Vti1a/Vti1b double-deficient mice, whole-cell patch-clamp recordings demonstrated an 80–90% reduction in post-stimulation-evoked neurotransmitter release and a 65% reduction in the frequency of spontaneous fusion events (Emperador-Melero et al., 2018). Interestingly, the levels of exocytotic proteins SNAP25 and Munc13-1, Synaptobrevin-2, Stx1, and Munc18-1 were reduced; and the delivery of SNAP25 and DCV cargoes to the axon was reduced, and these molecules accumulate in the Golgi apparatus (Emperador-Melero et al., 2018). These secretion defects can be almost completely rescued by exogenous expression of Vti1a (Emperador-Melero et al., 2018). Thus, these evidences seem to suggest that Vti1a may mediate vesicle fusion by affecting horizontal synapses of associated exocytotic proteins as well as upstream events of apical vesicle secretion. However, whether Vti1a is directly involved in the fusion of synaptic vesicle membranes remains unknown. Deletion of Vti1a impairs spontaneous high-frequency glutamate release, which exerts pathogenic effects (Crawford et al., 2017). Vti1a knockdown results in reduced spontaneous SV fusion (Ramirez et al., 2012; Crawford et al., 2017). Vti1a exhibits robust trafficking under neuronal resting conditions, which selectively maintains spontaneous neurotransmitter release (Ramirez et al., 2012). In addition, studies have shown that synaptotagmin-11 inhibits spontaneous release primarily by inhibiting Vti1a-containing vesicles (Li et al., 2021). The evidences suggest that spontaneous synaptic transmission is independent of evoked synaptic transmission and that Vti1a is a joint regulator of this process. However, the downstream pathways of Vti1a-mediated regulation of vesicle activity and the corresponding molecular mechanisms remain unclear.

Vti1a also acts as a key regulator of the postsynaptic efficacy of spontaneous synaptic transmission (Figure 2; Crawford et al., 2017). Eukaryotic elongation factor-2 kinase (eEF2K) is a Ca^2+^/calmodulin-dependent serine/threonine kinase that is essential for regulating the elongation in protein translation (Heise et al., 2014). Its inactivation leads to the dephosphorylation of postsynaptic eEF2, which inhibits brain-hippocampus-derived neurotrophic factor (BDNF) protein synthesis, affecting neuronal activity (Verpelli et al., 2010; Suzuki and Monteggia, 2020). Loss of Vti1a impairs eEF2K signaling, resulting in decreased eEF2 phosphorylation (Crawford et al., 2017).

### Regulation of Neural Development

Normal neural development involves various stages of synaptic development and various links of neuronal connections, and this process is regulated by a variety of proteins, such as synaptic cell adhesion molecules and SNARE (Hepp and Langley, 2001; Ko, 2012). Major neurodevelopmental events associated with SNAREs include progenitor cell proliferation, neurite outgrowth, neuronal migration, synapse formation and transmission, and neurodegeneration (Hepp and Langley, 2001; Kimura et al., 2003; Wang and Tang, 2006). Double deletion of the *VTI1A/1B* gene impairs neuronal process outgrowth and projection leading to abnormal neuronal differentiation or maturation (Sokpor et al., 2021). For example, axonal loss and neurodegeneration in peripheral ganglia were observed in *VTI1A/1B* double-deficient mouse embryos, resulting in severely impaired neuronal development (Kunwar et al., 2011). In addition, a *VTI1A/1B* null mutation in mice resulted in a dysplastic cortex in late embryos (Sokpor et al., 2021). However, individual deletions of Vti1a or Vti1b are tolerated in mice, whereas simultaneous deletions cause extensive neurodegeneration and lethal effects (Atlashkin et al., 2003; Kunwar et al., 2011). Although the Vti1a deficiency is not fatal, it adversely affects neuronal development and metabolism (Kunwar et al., 2011).

### Regulation of Autophagy

Autophagy is a cellular degradation pathway which autophagosomes fuse with lysosomes to form autophagolysosomes and degrade their contents (Iriondo et al., 2021; Gubas and Dikic, 2022). The autophagy pathway involves a series of membrane fusion events and requires the participation of SNARE proteins, such as Syn17, VAMP3, VAMP7, YKT6, Vti1 (Tian et al., 2021). For example, mutation of Vti1p in yeast disrupts autophagosome-vacuole fusion (Ishihara et al., 2001). In mammals, the abnormality of Vti1a and Vti1b affects the formation of autophagosomes (Figure 1; Lu et al., 2013; Chou et al., 2021). Mutations in the *CHMP2B* gene can cause frontotemporal dementia, the pathogenicity of which is primarily thought to be the result of autophagy-endolysosomal dysfunction (Deng et al., 2022; Roos et al., 2022). Stx13, as a strong genetic modifier of CHMP2B, is involved in phagocyte maturation and affects autophagosome formation (Lu et al., 2013). Knockdown of Stx13 or its binding partner Vti1a leads to accumulation of the autophagy marker LC3, affects autophagosome maturation, and blocks autophagic flux (Lu et al., 2013). Although studies have shown that regulation of Vti1a and Vti1b can inhibit phagocytosis in phagocytes and may play a regulatory role in the process of autophagy (Cai et al., 2011). However, the specific mechanism by which Vti1a regulates the autophagy process remains unclear.

### Regulation of Unconventional Extracellular Transport in Neurons

The K channel interacting proteins (KChIPs) belong to the neuronal calcium receptor family and assemble into a natural complex with the α subunit of the voltage-gated Kv4 potassium channel, encoding A-type K^+^ current to regulate neuronal excitability (Bähring, 2018; Kise et al., 2021). The specific assembly contributes to forming and stabilizing voltage-gated potassium channel tetramers and increases channel transport to the cell membrane surface (Alfaro-Ruíz et al., 2020). For example, siRNA-mediated knockdown of Vti1a or VAMP7 inhibited Kv4/KChIP1 transport to the Neuro2A cell membrane (Flowerdew and Burgoyne, 2009). The VAMP7/Vti1a SNARE complex controls an unconventional traffic route to the cell surface used by KChIP1 and Kv4 potassium channels (Figure 2; Flowerdew and Burgoyne, 2009). These evidences suggest that neurons depend on an unconventional exocrine pathway of Vti1a and VAMP7 for Kv4/KChIP1 trafficking. However, the nature of this pathway and the membrane transport mechanism still need to be explored further.

## VTI1A and Neurological Disorders

Studies have shown that Vti1a plays a substantial role in some neurological disorders (Table 1); Vti1a gene variants and fusion transcripts with adjacent genes are involved in gliomas (Wang et al., 2017); Vti1a plays a key role hepatic encephalopathy (Popek et al., 2018) by regulating spontaneous neurotransmitter transmission. Furthermore, Vti1a is involved in ALS, but its specific role is unclear (Nagao and Hayashi, 2010).

**TABLE 1:** The role of Vti1a in neurological disorders.

Type of nervous system disorders	Abnormal Vti1a	Mechanisms of Vti1a involvement	References
Glioma	Fusion of the *VTI1A* and *TCF7L2* gene/*VTI1A* SNP variant rs11196067	Regulating the Wnt signaling pathway to promote the progression of Glioma	Kinnersley et al., 2015; Wang et al., 2020
Hepatic encephalopathy	Decreased expression	Inhibiting spontaneous transmission to promote the progression of Parkinson’s disease	Popek et al., 2018
Amyotrophic lateral sclerosis	Decreased expression	–	Nagao and Hayashi, 2010

### Glioma

Vti1a forms fusion products with adjacent genes in human cancer tissues, and Vti1a variants are associated with the development of various cancers (Table 2; Gao et al., 2016; Wang et al., 2016, 2017; Zhang et al., 2018; Tsuge et al., 2019). A previous study identified *VTI1A* as one of the susceptibility genes for glioma in European populations (Kinnersley et al., 2015). Interestingly, in 473 Chinese glioma, the *VTI1A* single nucleotide polymorphism (SNP) variant rs11196067 was significantly associated with its risk, suggesting that *VTI1A* variants might increase the susceptibility of individuals to glioma (Wang et al., 2017). In addition, the results of a meta-analysis of four genome-wide association studies on glioma revealed that the *VTI1A* SNP variant rs11196067 was a susceptibility gene in glioma (Kinnersley et al., 2015).

**TABLE 2:** The mutation/variant of Vti1a in different cancers.

Cancer type	Vti1a mutation/variant	Mechanism	References
Glioma	*VTI1A-TCF7L2* gene fusion/*VTI1A* variant rs11196067	Regulating the Wnt signaling pathway	Harterink et al., 2011; Kinnersley et al., 2015; Wang et al., 2020
Liver cancer	*VTI1A-CFAP46* gene fusion	Regulating autophagy	Tsuge et al., 2019
Lung cancer	*VTI1A* variant rs7086803	Hypomethylation of *VTI1A*	Wang et al., 2016
Colorectal cancer	*VTI1A-TCF7L2/*rs12241008	Regulating the Wnt signaling pathway	Davidsen et al., 2018; Zhang et al., 2018
Breast cancer	rs7903146, rs7904519	–	Zhang et al., 2018

In GBM of glioma, genomic and transcriptome sequencing revealed gene fusions in approximately 30–50% of patients (Shah et al., 2013). The transcription factor 7-like 2 (TCF7L2) gene is located on chromosome 10q25.2, about 131 kb downstream of the *VTI1A* gene (Zhang et al., 2018). Several variants were identified in the *VTI1A-TCF7L2* fusion gene region that were associated with tumor risk, including glioma (Zhang et al., 2018). Surprisingly, the *VTI1A-TCF7L2* gene fusion transcripts were detected in the tumor tissue and plasma of GBM (Wang et al., 2020). The *TCF7L2* gene product is a transcription factor containing a high mobility group cassette that plays an essential role in the Wnt signaling pathway (Su et al., 2015; Torres-Aguila et al., 2022). The Wnt signaling pathway has been shown to play an important role in the progression of glioma by affecting the differentiation, proliferation, migration, and apoptosis of neural cells (Tompa et al., 2019; Pei et al., 2022). Recent studies have shown the presence of glioma stem cells in GBM (Ma et al., 2020). Wnt/β-catenin is a major signaling pathway in brain development, regulating the self-renewal and differentiation of neural stem and progenitor cells (Tompa et al., 2018). Studies have shown that fusion of the *VTI1A* and *TCF7L2* genes, encoding a VTI1A-TCF4 fusion protein containing truncated TCF4, regulates the Wnt signaling pathway and participates in colorectal cancer development (Davidsen et al., 2018). The Vti1a-TCF4 fusion product may interfere with oncogenic signaling in the brain to promote gliomagenesis through a similar mechanism. In addition, the VTI1A gene encodes the v-SNARE protein, which mediates the transport of vesicles from the endosome to the TGN. A sorting molecule, the Wnt-interacting receptor, was found to circulate from the plasma membrane to the Golgi through early endosomes (Harterink et al., 2011). This process may be regulated by Vti1a (Harterink et al., 2011). In short, abnormal Vti1a may affect Wnt-interacting receptor signaling or promote tumor cell migration in an as yet undetermined manner, but the exact mechanism is unclear.

### Others

Hepatic encephalopathy is a complex neuropsychiatric syndrome, usually resulting from acute or chronic liver failure, and is associated with decreased excitatory neurotransmission (Albrecht et al., 2010; Palomero-Gallagher and Zilles, 2013). Impaired release of synaptic neurotransmitter transmission with concomitant reduction of kinesin Vti1a was observed in mice with azomethane-induced hepatic encephalopathy (Popek et al., 2018). This alteration may be related to inefficient Vti1a protein recruitment and impaired SV transport to neurotransmitter release sites (Popek et al., 2018). Another study on the expression of glycogen synthase kinase 3beta in the spinal cord of amyotrophic lateral sclerosis (ALS) reported decreased TGN GSK3beta expression in motor neurons, as well as Vti1a (Nagao and Hayashi, 2010). However, the exact mechanism of action is still unknown.

## Prospective

In conclusion, Vti1a is a critical vesicular protein that primarily regulates neurosecretory. It has essential roles in spontaneous neurotransmitter transmission, neuronal autophagy, neural development, and unconventional extracellular transport. Furthermore, Vti1a forms transcriptional complexes with adjacent genes, and Vti1a variants play critical roles in glioma. Therefore, Vti1a is highly relevant in neurological diseases and presents substantial research potential. For example, one study found that the Vti1a gene transcript could be detected in tumor tissue and serum of GBM (Wang et al., 2020). This evidence suggests that Vti1a has the potential to be a candidate biomarker for GBM. However, the sample size of that particular study was small, and further research is needed to confirm the results.

Nevertheless, several issues still need to be resolved. For example, how do *VTI1A* variants or mutants combine with gene fusion products or drive tumorigenesis on their own? As a protein involved in spontaneous neurotransmission, what is the relationship between VAMP7 and Vti1a? How do these proteins function in complex neurotransmission? Increased understanding of the function and regulatory mechanisms of Vti1a might provide potential therapeutic targets for related diseases.

## Author Contributions

FT and DX: conceptualization. FT, DX, XZ, and XL: software. FT, DX, ZZ, and XL: resources. FT and JF: writing–original draft preparation. FT, DX, XZ, ZZ, and XL: writing-review and editing. XZ and XL: visualization. DX and XL: supervision. All authors read and approved the final manuscript.

## Conflict of Interest

The authors declare that the research was conducted in the absence of any commercial or financial relationships that could be construed as a potential conflict of interest.

## Publisher’s Note

All claims expressed in this article are solely those of the authors and do not necessarily represent those of their affiliated organizations, or those of the publisher, the editors and the reviewers. Any product that may be evaluated in this article, or claim that may be made by its manufacturer, is not guaranteed or endorsed by the publisher.

## Abbreviations

Vti1a: Vesicle transport through interaction with t-SNAREs 1A
SNARE: N-ethylmaleimide -sensitive factor attachment protein receptor
VAMP: vesicle-associated membrane protein
Syb: Synaptobrevin
Stx: Syntaxin
TGN: *trans-*Golgi network
SV: synaptic vesicle
DCV: dense core vesicle
eEF2K: eukaryotic elongation factor-2 kinase
BDNF: brain-hippocampus-derived neurotrophic factor
KChIP: K channel interacting proteins
TCF7L2: The transcription factor 7-like 2
SNP: single nucleotide polymorphism
GBM: glioblastoma
NMDAR: N-methyl -D-aspartate receptor
AMPA: amino-3-hydroxy-5-methyl-4-isoxazolepropionic acid.
